# Comparative analysis of short-term efficacy between robot-assisted retrograde drilling and arthroscopic microfracture for osteochondral lesions of the talus

**DOI:** 10.3389/fsurg.2024.1404513

**Published:** 2024-05-27

**Authors:** Xiao Qiang Chen, Jianquan Liu, Tianyu Wang, Zhe Zhao, Yongsheng Li, Xiang Yu Cheng, Wencui Li

**Affiliations:** Hand and Foot Surgery Department, Shenzhen Second People's Hospital, The First Hospital Affiliated to Shenzhen University, Shenzhen, China

**Keywords:** osteochondral lesions of the talus (OLT), arthroscopic microfracture, robot assisted navigation-retrograde drilling, Foot and Ankle, Talus - injuries

## Abstract

**Objective:**

To investigate the short-term clinical efficacy of robot-assisted retrograde drilling and arthroscopic microfracture for osteochondral lesions of the talus (OCLT).

**Methods:**

This study was divided into two groups: experimental group: robot-assisted retrograde drilling group; control group: arthroscopic microfracture group. A total of 6 OCLT patients who were treated with robot navigation-assisted retrograde drilling and 10 OCLT patients who were treated with arthroscopic microfracture between October 2020 and October 2021 were retrospectively analyzed. There were 11 males and five females, with a mean age of 36 years. The patients were followed up for 6–12 months to compare the changes in the OCLT lesion area by magnetic resonance imaging (MRI), visual analogue scale/score (VAS) and American Orthopedic Foot and Ankle Society score (AOFAS) before and after surgery.

**Results:**

All 16 patients were followed up for an average of 8 months, and no complications such as joint infection, nerve injury, or active bleeding occurred during the follow-up period. Only one patient suffered discomfort involving transient postoperative pain in the operative area, but did not experience long-term numbness or chronic pain. Postoperative MRI revealed that none of the patients had severe signs of osteonecrosis, osteolysis or cystic changes of the talus, with lesion areas smaller than those before surgery. The difference was statistically significant (*P *< 0.01). The patients in the experimental group showed a more significant improvement in the last 3 months than in the first 3 months of the follow-up period. At the last follow-up, the VAS score was 3 points in the experimental group and 2.2 points in the control group, and the AOFAS score was 88.6 points in the experimental group and 88 points in the control group, all of which were significantly higher than those before operation, and the differences were statistically significant, but there was no statistically significant difference between the groups.

**Conclusion:**

Both robot navigation-assisted retrograde drilling and arthroscopic microfracture for bone marrow stimulation (BMS) to treat OCLT in all patients obtained satisfactory effects in the short term. In addition, the follow-up revealed that with excellent efficacy and few complications, robot navigation-assisted retrograde drilling was safe and minimally invasive, and greatly reduced operative time. Consequently, robot navigation-assisted retrograde drilling for BMS was a safe and effective procedure for the treatment of OCLT.

## 1 Background

Osteochondral lesions of the talus (OCLT) are lesions involving the articular cartilage and subchondral bone of the talus, most of which are associated with chronic micro-injuries following ankle trauma and are prevalent in the athletic population (1). As previously reported, approximately 50%–73% of patients already have OCLT at initial diagnosis of acute ankle sprain (2, 3) and then develop typical clinical and imaging manifestations over time. According to the staging method proposed by Berndt and Harty, OCLT can be classified into four grades based on x-ray findings (4). In the past, severe grade III and IV OCLT were mainly treated by surgery. However, controversy remained concerning the treatment of grade I and II OCLT in the early and middle stages, and specific treatment attempts also varied. Recent studies concluded that conservative treatment was not effective for OCLT, even if the injury was mild, because of the difficulty of repairing the articular cartilage after injury, combined with a lack of blood supply from the talus itself (1). In recent years, it has been recommended that even minor grade I or II injuries should be treated by surgery as much as possible, or that the indications for surgery should be relaxed in order to allow early repair of cartilage damage. As a result, methods such as incisional or arthroscopic microfracture and retrograde drilling for bone marrow stimulation (BMS) are also widely applied (5). Minimally-invasive surgery is always the common pursuit of doctors and patients under the premise of ensuring complete treatment of lesions. Therefore, micro-fractures, cartilage or bone transplantation assisted by ankle arthroscopy and the use of surgical robots has emerged for the treatment of OCLT. In this paper, a novel surgical procedure is introduced from the perspective of surgical robots involved in the minimally-invasive treatment of early OCLT and the experience and gains of our team are reported.

The application of surgical robots in the field of ankle surgery has rarely been reported. It is believed that surgical robots will be more and more involved in foot and ankle surgery and even orthopedic surgery with further development of the technology and concept. Thus, the application of robot-assisted surgical treatment for early OCLT was expounded in this paper.

## 2 Patients and methods

### 2.1 General information

The clinical data of six patients who were treated by “robot navigation-assisted(TINAVI) retrograde drilling” and 10 patients who were treated by “arthroscopic(LINVATEC) microfracture” by the same team at our department between October 2020 and October 2021 were retrospectively included. This study was divided into two groups: experimental group: robot-assisted retrograde drilling group; control group: arthroscopic microfracture group. The patients comprised 11 males and five females, aged from 25 to 50 years, with a mean age of 36 years and disease course from 6 months to 2 years. All patients had a clear history of trauma and repeated multiple sprains of the ankle joint, with varying degrees of activity restriction and positive results for the ankle drawer test and inversion stress test on the affected side. Imaging by x-ray, computed tomography (CT) plain scan, 3D reconstruction and magnetic resonance imaging (MRI) examination of the ankle joint performed before surgery revealed no obvious signs of fracture of the talus but there were osteochondral injuries of different sizes and degrees, which all supported the clinical diagnosis of OCLT. The indications of retrograde drilling by the robot surgery should be: (1) The patient had ankle pain, and MRI or CT showed clear damage of talus cartilage. (2) Preoperative MRI or CT or intraoperative arthroscopy revealed that the osteochondral surface of the talus was intact, and subchondral lesions were the main problem and the main root cause of the patient's symptoms, which was the Helple stage of osteochondral injury of the talus. (3) Patients with a definite diagnosis of OCLT based on preoperative MRI, with a lesion diameter ≤15 mm or area ≤150 mm^2^. The indications of arthroscopic microfracture should be: (1) The patient had ankle pain, and MRI or CT showed significant damage to the talus cartilage. (2) Preoperative MRI or CT or intraoperative arthroscopy showed that the osteochondral surface of the talus was incomplete, with lacerations or dissociations. Preoperative MRI confirmed the diagnosis of OCLT in patients with lesion diameter ≤15 mm or area ≤150 mm^2^.

### 2.2 Inclusion and exclusion criteria

The inclusion criteria were (1) patients with a definite diagnosis of OCLT based on preoperative MRI, with a lesion diameter ≤15 mm or area ≤150 mm^2^. (2) Patients undergoing preoperative physical examination with or without chronic ankle instability. (3) Those with good compliance who could be followed up as scheduled after surgery.

The exclusion criteria were (1) patients with contraindications to surgical intervention, (2) those with definite fracture, bone disease or infection near the ankle joint, (3) those with a previous history of ankle surgery on the affected side or who were participating in other clinical trials and (4) those who were unwilling or unable to complete follow-up visits as scheduled.

## 3 Methods

### 3.1 Robot navigation-assisted retrograde drilling

**Figure 1:**
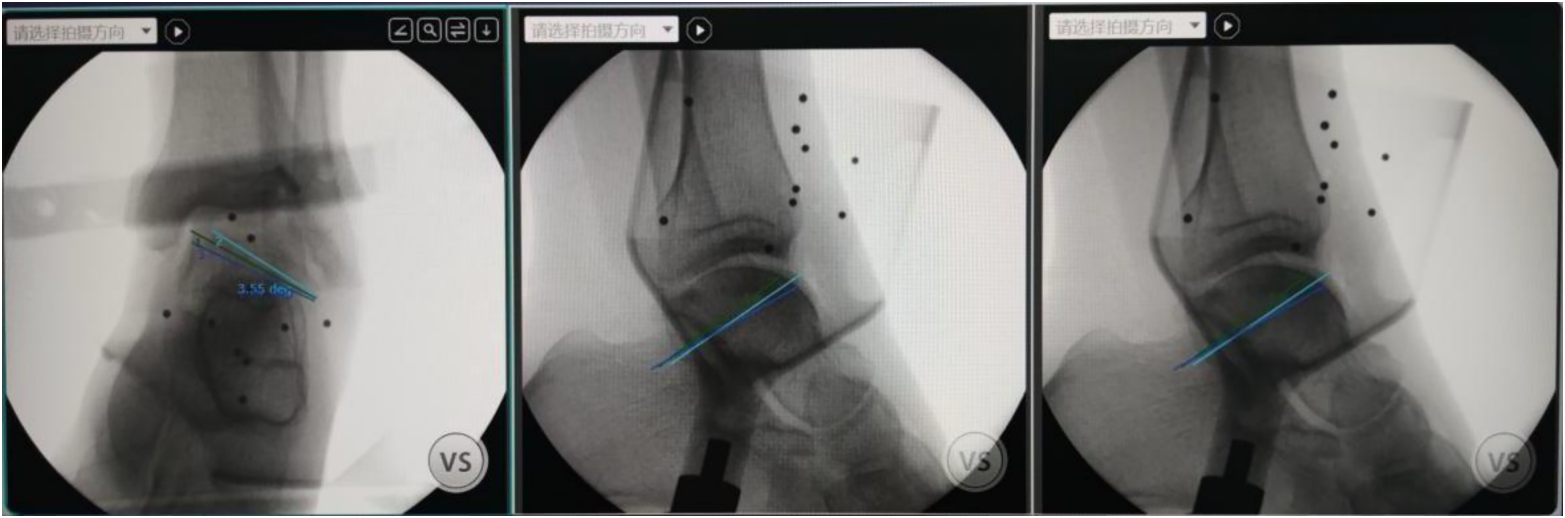
Three optimal paths for retrograde drilling were marked on the computer based on the overall positioning of the ankle joint identified by the robot.

**Figure 2:**
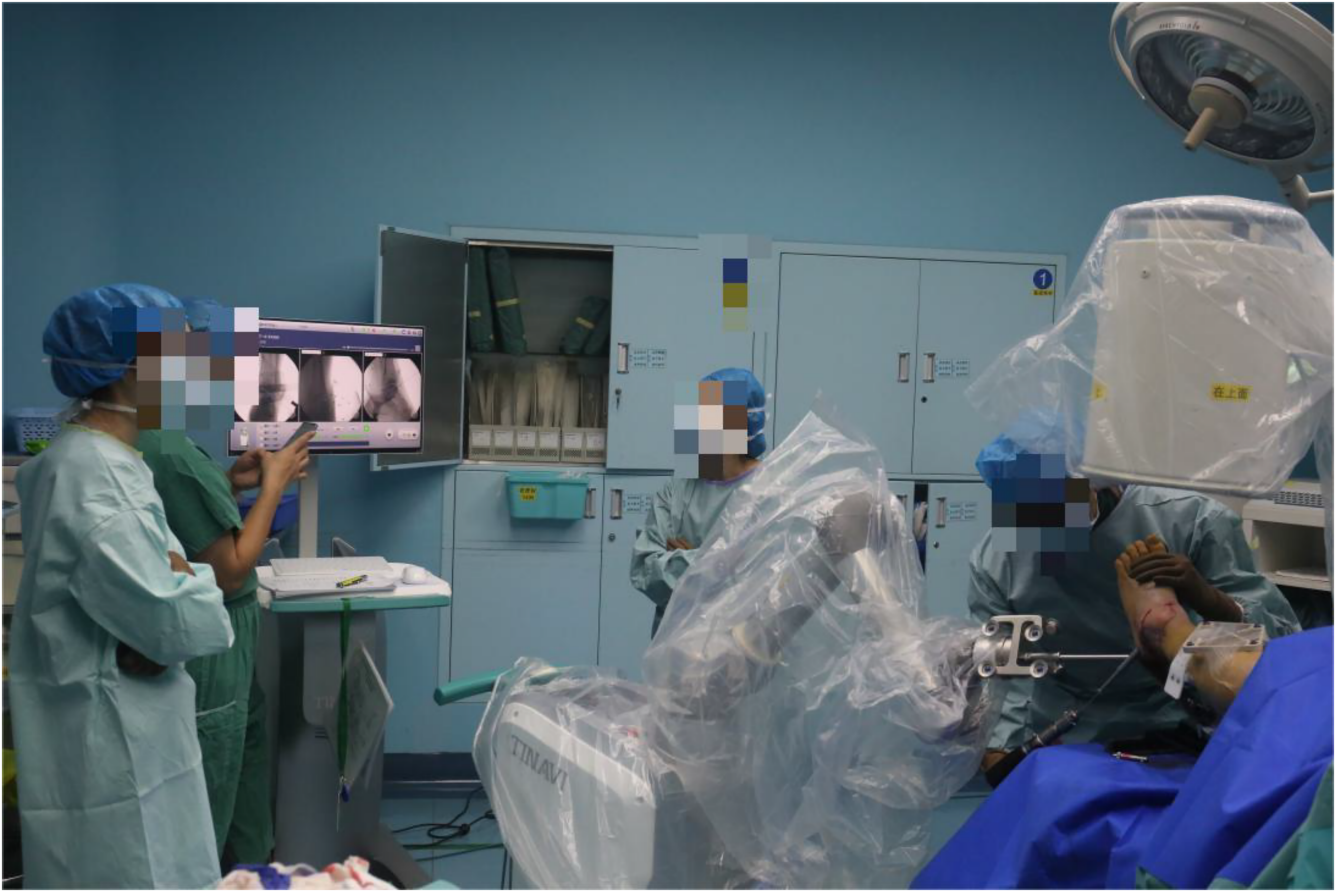
With the aid of the robot, retrograde drilling through the posterior lateral approach according to the designed paths.

All the surgeries were performed by the same team of foot and ankle surgeons and the affected ankle was subjected to appropriate traction using a traction frame with the patient in the supine position under epidural anesthesia or general anesthesia + nerve block anesthesia. The anteromedial approach to the ankle joint was used as the observation approach to insert the arthroscopic lens. The anterolateral approach was used as the operation approach under guidance of the arthroscopic lens inserted to probe the ankle joint and clear hyperplastic synovium, bony redundancy and free bodies, etc. Additionally, the OCLT lesion was probed under microscopic vision to ensure that the cartilage surface of the talus was intact and free from breakage, defects, freeing and separation, etc. and the integrity of the anterior talofibular ligament was investigated. The arthroscope was then removed, and the robot system was placed in position and calibrated to locate the key points of the ankle joint. During the operation, the ankle joint was photographed in frontal, lateral and oblique views using a fluoroscope, and 3–8 optimal paths for retrograde drilling were marked on the computer based on the overall positioning of the ankle joint identified by the robot (Figure 1), then retrograde drilling was performed under robot navigation. If the lesion was located in the medial talus, the posterior lateral approach was selected as the drilling approach (Figure 2), and the anterior medial approach was selected if the lesion was located in the lateral talus. After drilling, the positive and lateral views of the ankle joint were observed under a fluoroscope to ensure the accuracy and depth of each path. The anchor-nail suture (Smith & Nephew Smith) was performed under fluoroscopy if the anterior talofibular ligament needed to be repaired or reconstruction. Whether it is necessary to anchor and renew the ligament needs to be combined with these 4 evaluation criteria to make a decision. The evaluation criteria is: (1) Preoperative anterior drawer tests of the ankle joint. (2) MRI to assess ATFL integrity. (3) ATFL tension and integrity were examined by arthroscopy. (4) Medical history collection. Whether the ligament needs to be repaired or reconstructed depends on the following criteria. Criteria for ATTL repair: There was no history of recurrent ankle sprains within 1 year, MRI indicated the presence of ligament morphology, and the ligament was detected under arthroscopy but appeared to be relaxed. Criteria for ATTL reconstruction: The patient had a 1-year history of recurrent ankle sprain, MRI showed no ligament shape, no ligament was found under arthroscopic exploration, and only scar tissue structure around the ligament existed.

### 3.2 Arthroscopic microfracture

All surgeries were performed by the same team of foot and ankle surgeons and the affected ankle was subjected to appropriate traction using a traction frame with the patient in the supine position under epidural anesthesia or general anesthesia + nerve block anesthesia. The anteromedial approach to the ankle joint was used as the observation approach to insert the arthroscopic lens. The anterolateral approach was used as the operation approach under guidance of the arthroscopic lens inserted to probe the ankle joint and clear hyperplastic synovium, bony redundancy and free bodies, etc. Additionally, the OCLT lesion was probed and the cartilage and subchondral bone were cleaned, measured and photographed, and microfracture was performed on the bone with a suitable type of microfracture cone, subject to visible bone marrow fluid overflow. The anchor-nail suture was performed under fluoroscopy if the anterior talofibular ligament needed to be repaired or reconstruction. The evaluation criteria were the same as in the robot-assisted retrograde drilling surgery group. Techiniques of ATFL reconstructive surgery: (1) Part of peroneal short tendon was transplanted; (2) The transplanted tendon was fixed in the distal fibula and lateral talus with interface screws; (3) Hold the ankle joint in a neutral position when fixing the tendon. Techiniques of ATFL repair surgery: (1) Ensure that the quality of residual ATFL is sufficient for suture; (2) Anchor implantation point should be at ATFL anatomical footprint as far as possible; (3) Hold the ankle joint in a neutral position while tying and stitching.

### 3.3 Postoperative management

Regardless of the surgical procedure, the affected ankle joint was fixed in a neutral position for 2 weeks immediately after surgery, and postoperative symptomatic treatment such as pain relief, cold therapy and physiotherapy was given. The patients were followed up regularly at 1, 3, and 6 months after surgery, and MRI of the ankle joint was repeated for changes in OCLT area (expressed as a percentage of postoperative area reduction compared to preoperative area), and scales such as Visual Analogue Scale/Score (VAS) and American Orthopedic Foot and Ankle Society score (AOFAS).

### 3.4 Statistical analysis

SPSS 25.0 software was adopted for statistical analysis, and quantitative data are expressed as mean ± s. Quantitative data were subject to normality and homogeneity of variance tests, and *t*-tests were applied for comparison of data meeting the definitions of normality and homogeneity of variance. Data not meeting normality and homogeneity of variance were analyzed using the Mann-Whitney *U* test. Count data are expressed as the number of cases (%) and tested using the Chi-square test or non-parametric test. Regression analysis effect values and 95% confidence intervals (CI) were applied to illustrate the core results. *P *< 0.05 was considered statistically significant.

## 4 Results

All patients were diagnosed with OCLT, with a mean lesion size of 5.1 × 7.2 mm on preoperative MRI. Sixteen patients were followed up for 6–12 months, with a mean follow-up period of 8 months. No patient suffered complications such as joint infection, nerve injury, or active bleeding during follow-up, while one patient suffered from discomfort involving transient postoperative pain in the operative area. All patients showed better postoperative VAS scores, AOFAS scores, and reduced lesion areas on MRI than those before operation, with the differences being statistically significant (*P *< 0.01). Arthroscopic ATFL repair was performed in 4 of the 6 robot-assisted surgery groups, and arthroscopic ATFL repair was performed in 5 of the 10 arthroscopic microfracture groups, and arthroscopic ATFL reconstruction was performed in 2 of the 10 arthroscopic microfracture groups.

**Figure 3:**
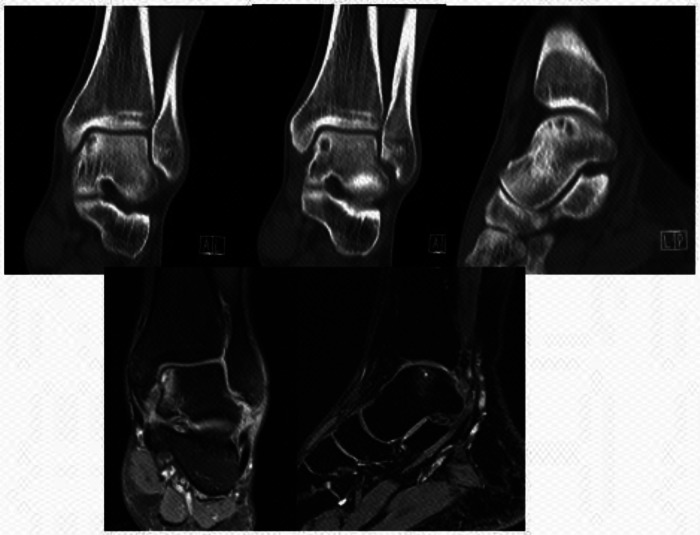
Preoperative CT and MRI image data.

**Figure 4:**
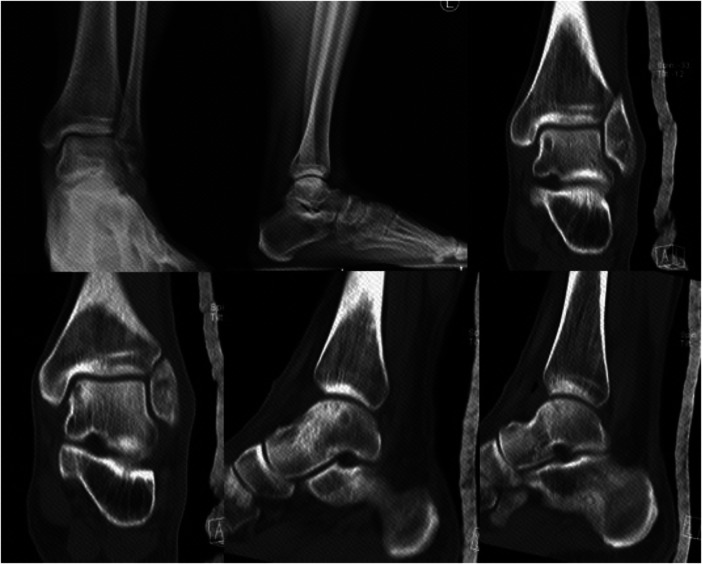
Immediately CT after surgery showed the ideal paths of retrograde drilling surgery.

**Figure 5:**
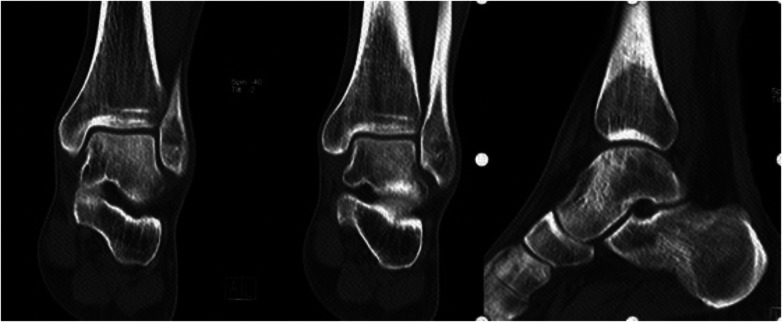
Talus cartilage injury was repaired and subchondral bone filling was full 2 months after surgery.

**Figure 6:**
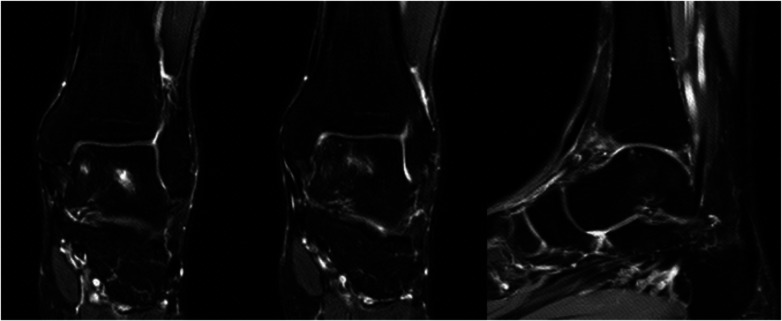
MRI review showed that the talus cartilage was repaired satisfactorily and the edema area was significantly reduced 5 months after surgery.

The following is a case of OCLT located on the medial side of the talus. Preoperative CT and MRI confirmed that the OCLT was located on the medial side of the talus, with an area of about 15 mm^2^ (Figure 3). Intraoperative arthroscopic exploration did not show surface collapse, separation or rupture of the talus cartilage, so the patient adopted robot-assisted retrograde drilling surgery. Reexamination of CT immediately after surgery (Figure 4) showed the ideal paths of retrograde drilling surgery, which accurately reached the damaged area of talus cartilage. Reexamination of CT 2 months after surgery showed that talus cartilage injury was repaired and subchondral bone filling was full (Figure 5). MRI review 5 months after surgery showed that the damaged site of the talus cartilage was repaired satisfactorily, the edema area was significantly reduced compared with that before surgery, and the pain symptoms of the patient had completely disappeared (Figure 6).

### 4.1 OCLT lesion location

Of the 16 patients included in the study, seven patients had lesions located in zone 4 (medial), one in zone 7 (posterior medial) four in zone 3 (anterolateral) and the remaining four patients had lesions located in zone 1 (anteromedial). No severe signs of local necrosis, osteolysis or cystic degeneration of the talus were observed in any of the patients (Tables 1, 2).

**Table 1:** Basic information of patients who underwent robot navigation-assisted retrograde drilling.

	Gender	BMI (kg/m^2^ (kg/m)	Lesion site (block box)	Area (mm^2^)	Staging	ATFC condition	Symptom duration (month)
1	Male	19.7	4	37.3	2		8
2	Male	23.8	4	25.8	2		7
3	Female	20.6	1	41.5	2		13
4	Male	24.7	4	28.6	2		6
5	Female	22.5	3	36.0	2		9
6	Male	24.3	1	43.2	1		7

**Table 2:** Basic information of patients who underwent arthroscopic microfracture.

	Sex	BMI (kg/m)	Lesion site (block box)	Area (mm^2^)	Staging	ATFC condition	Symptom duration (month)
1	Female	23.7	6	30.0	2		12
2	Male	32.0	4	32.6	3		11
3	Male	24.9	7	40.8	2		6
4	Male	22.2	1	44	1		7
5	Female	20.8	4	50.4	3		14
6	Female	19.8	4	26.4	2		10
7	Male	22.0	3	28.5	3		5
8	Female	27.4	3	45.0	2		15
9	Male	26.5	4	48.8	2		12
10	Male	20.0	1	32.2	1		11

### 4.2 VAS score

Six patients who underwent robot navigation-assisted retrograde drilling had effectively improved VAS scores at 1, 3, and 6 months after surgery (*P *< 0.05), and the difference was statistically significant. It was noted in the follow-up that 10 patients who underwent arthroscopic microfracture surgery had lower VAS scores at 1, 3 and 6 months after surgery than those before surgery, with the difference being statistically significant. However, there was no significant difference between the experimental group and the control group (Table 3).

**Table 3:** VAS scores of each group of patients before and after surgery.

	Before surgery	1 month after surgery	3 months after surgery	6 months after surgery
Control group	4.5	3.7	3.5	2.2
Experimental group	4.8	4.5	4.3	3.0
*P* value in control group before and after surgery		0.25	0.0625	0.002
*P* value in experimental group before and after surgery		0.125	0.125	0.0043
Difference between groups in the same time period	0.604	0.0633	0.1089	0.651

### 4.3 AOFAS scores

Among the 16 patients included in the study, six patients who underwent robot navigation-assisted retrograde drilling exhibited a gradual increase in their AOFAS scores at the 1, 3, and 6 month postoperative follow-up visits compared with those before surgery. Ten patients who underwent arthroscopic microfracture showed gradual increases in their VAS scores at 1, 3 and 6 months after surgery, and these differences were statistically significant. However, there was no statistically significant difference between the two groups (Table 4).

**Table 4:** AOFAS scores of each group of patients before and after surgery.

	Before surgery	1 month after surgery	3 months after surgery	6 months after surgery
Control group	81.6	84.2	86.3	88
Experimental group	82.0	83.3	85.5	88.6
*P* value in control group before and after surgery		0.0277	<0.0001	<0.0001
*P* value in experimental group before and after surgery		0.0067	0.0076	0.0047
Difference between groups in the same time period	0.7734	0.4811	0.2504	0.7107

### 4.4 Changes in OCLT lesion area on postoperative MRI compared with those before surgery

Of the 16 patients followed up, six patients in the experimental group exhibited smaller lesion areas than those before surgery based on imaging data at 1, 3 and 6 months and the patients in the experimental group exhibited more significant improvement in the second 3 months than in the first 3 months. Ten patients in the control group had smaller lesion areas than those before surgery at the 1, 3 and 6 month follow up. However, the control group showed more significant changes in the first 3 months than in the second 3 months, which differed from the experimental group (Table 5).

**Table 5:** Percentage change (%) in lesion area (mm^2^) on MRI of each group before and after surgery.

	Before surgery	1 month after surgery	3 months after surgery	6 months after surgery
Control group	35.4	32.7 (7.6%)	26.6 (24.9%)	8.2 (76.8%)
Experimental group	37.9	32.0 (15.6%)	24.0 (36.7%)	10.4 (72.6%)
*P* value in control group before and after surgery		<0.0001	<0.0001	<0.0001
*P* value in experimental group before and after surgery		0.003	0.0007	<0.0001
Differences between groups in the same time period	0.0717	0.4596	0.002	0.0031

Experimental group: robot-assisted retrograde drilling group; Control group: arthroscopic microfracture group.

## 5 Discussion

Flick and Gould (6) revealed that in 98% of cases, OCLT was associated with traumatic events, especially chronic, microscopic and recurrent trauma, with the most common being ankle sprain. In many patients, OCLT is accompanied by symptoms such as ankle ligament relaxation, injury and even absorption (6). In fact, a study on the treatment of OCLT involving 1,804 physicians in 79 different countries around the world demonstrated that most surgeons did not treat OCLs alone, but rather included ankle ligament repair or reconstruction in their treatment (7).

Researchers focusing on OCLT lesions have found that if the surface of the talus fornix joint was divided into block boxes, most injuries occurred in zone 4 (i.e., the medial zone) (8). The talus has no muscular attachment and is covered with cartilage over 60% of its surface, which provides a good contact surface for movement and weight-bearing in the ankle joint, especially the tibial talar joint, but also greatly limits the blood supply to the talus itself and the cartilage attached to it. As a result of these anatomical features once the talar bone or cartilage is damaged, it cannot effectively repair itself, regardless of whether the original blood supply is damaged or not. Traditionally, immobilization, physiotherapy, oral medication and joint injections are still adopted for the treatment of OCLT of grades I and II and surgical intervention is considered only after conservative treatment is not effective. However, conservative treatment of OCLT is often not effective due to the anatomical characteristics of the talus itself as described above. Nearly 50% of OCLT patients receiving conservative treatment have been reported to have a poor outcome or even failed treatment (9). Therefore, it is suggested that active surgical intervention be adopted for early to mid-stage OCLT, or corresponding surgical indications should be relaxed with the aim of facilitating lesion repair through surgery.

For surgical treatment, small area osteochondral injuries (generally ≤15 mm in diameter) are generally treated using procedures such as microfracture and retrograde drilling procedures for bone marrow stimulation (BMS) to promote healing of the bone wound, and the cartilage defect is repaired in the form of fibrocartilage at a later stage. BMS surgery for OCLT exhibits remarkable short-term efficacy. A study by Steman et al. found that 88% of patients resumed bracing movement after surgery (10). A study by Hurley et al. also demonstrated that 86.8% of patients receiving BMS surgery returned to normal exercise within an average of 4.5 months (11). Moreover, a retrospective study by Van Eekeren found that 76% of such patients also returned to normal exercise over a longer (mean 118 months) follow-up period (12), indicating that BMS still provided satisfactory results, although the effectiveness of BMS declined over the longer term. In a study involving 105 cases of ankle OCLT, Chuckpaiwong et al. noted that for all 73 patients with lesion diameters <15 mm, BMS achieved satisfactory results with VAS and AOFAS as indicators, but showed sharply reduced efficacy for patients with lesion diameters ≥15 mm. Furthermore, they specifically noted that BMS was not effective for lesions larger than 20 mm (13).

For OCLT involving large areas (namely, 15 mm or more in diameter or 150 mm2 or more in area), BMS has been proven to not be an effective treatment and to have a high risk of surgical failure. In fact, for patients with large injuries or cases in which the above treatments have failed, osteochondral grafting, such as cartilage grafts, bone grafts with periosteum, or chondrocyte implantation (the most common type being matrix-induced autogenous chondrocyte implantation, MACI) are applied to promote healing of the bone wound and ultimately repair the cartilage defect in the form of fibrocartilage or hyaline cartilage. Autogenous osteochondral implantation has been demonstrated to be effective in the treatment of large-area osteochondral defects, and can be used to repair the lesion with intact hyaline cartilage and subchondral bone. In a study involving 50 patients with large lesions, Shim et al. reported a case that after a mean follow-up period of 118 months, all 18 patients receiving autologous osteochondral grafts showed good efficacy, while in contrast all 32 patients receiving BMS exhibited unsatisfactory efficacy. They believed that the large lesion size was an important factor in the failure of the BMS procedure (14). A meta-analysis by Dexter et al. also indicated that autologous osteochondral grafts enabled patients to return to daily activities and physical activity earlier (15). However, this donor-site sacrifice procedure has extremely strict requirements on the donor site. Indeed, the greatest complication of this procedure is still observed at the donor site. Osteochondral tissue implantation from a non-weight-bearing area of the knee joint results in complications of slow recovery of the cartilage in the donor area, and pain and limitation of movement due to osteochondral destruction during this period (16). Osteochondral allograft transplantation can also be used, but widespread adoption is unlikely due to its susceptibility to rejection. Periosteal bone grafting was first proposed as an option for the repair of OCLT by Prof. Qinwei Guo and team and they reported that this procedure had no significant disadvantages compared with traditional osteochondral grafting, and satisfactory outcomes were achieved during the 5-year postoperative follow-up period (17), while similar conclusions were reached by a team in China in a subsequent study. However, it is considered that there is still a lack of large-scale clinical evidence to support this approach. For large osteochondral lesions, grafting is not the only technique available. Several surgical techniques have been proposed to avoid the donor-site morbidity. For example AMIC technique is widely use for this pathology. In a study involving 63 patients with large lesions, Ben et al. reported A-AMIC yielded clinical improvements at a minimum follow-up of 60 months in patients with symptomatic OLTs, with clinical improvement peaking in the first 2 years, followed by a plateau period (18).

In this study, both microfracture surgery and robot-assisted retrograde drilling achieved satisfactory short-term clinical efficacy, and the study results show that robot-assisted retrograde drilling surgery is a safe, effective, minimally invasive and innovative new surgical method. Surgical methods for osteochondral injury of talus are very diverse, among which microfracture and robot-assisted retrograde drilling have proved their effectiveness and superiority in clinical practice, and have also been widely reported in the field of foot and ankle surgery and sports medicine. The robot-assisted retrograde drilling procedure compared in this paper is a bold innovation in the mode of retrograde drilling surgery, but the principle is essentially the same as retrograde drilling. In this paper, there are indeed differences in the integrity of cartilage among patients in the two surgical methods, which in theory may cause deviations in the results, especially in terms of medium and long-term efficacy. However, in terms of short-term efficacy, there is no literature that clearly indicates that the integrity of surface cartilage may cause deviations in the results. The comparison of this study is the difference in short-term efficacy between the two surgical methods. The removal of cartilage did not bias the results of this study.

The MACI procedure is a safe and reliable treatment for OCLT, and a study on OCLT patients who underwent MACI, with a follow-up period ranging from 2 to 12 years, found that MACI showed satisfactory long-term effects, with most patients having significant increases in AOFAS scores and postoperative functional recovery after surgery. On the other hand, given the cost expenditure and actual results, MACI is not considered to have significant advantages over traditional procedures such as microfracture and autologous osteochondral implantation, and therefore is not recommended as the preferred treatment option for OCLT (16).

Surgical robots have been a hot spot for clinical innovation since their emergence, with the aim of integrating precise navigation and positioning of human anatomy with specific operational control (19). The concept of minimally-invasive surgery (MIS) has been constantly developed in various surgical specialties and has become an accepted surgical procedure to reduce patient pain since surgical robots were first involved in neurosurgical tumor resection in the form of computer-assisted positioning in 1985 and the first lumpectomy was performed in the same year (20). It was reported that in 2016, surgical robots were involved in more than 750,000 surgeries in the United States, mostly in urology and gynecology (19). Specifically in orthopedics, it was reported that in 1986, commercial surgical robots were first involved in improving the outcomes of total hip replacement and since then, the use of surgical robots has spread and they can now be found in traumatic fracture, spine, joint replacement, and other specialty surgeries following developments in the technology (21–23). Robot navigation-assisted retrograde drilling for OCLT allows for precise drilling to avoid the disadvantages of blindness and uncertainty of conventional retrograde drilling, so as to reduce the amount of fluoroscopy and thus shorten operative time. Further, with the aid of a robot, the drilling depth can be measured accurately to avoid the complications of cartilage surface destruction resulting from blind retrograde drilling, thereby effectively protecting the hyaline cartilage of the talus. However, robot navigation-assisted retrograde drilling also has some disadvantages. The lead surgeon must be proficient in the use of the navigation system due to its complex operation requirements. At the same time, accurate installation of the positioner of the robot navigation system and comprehensive capture of the signal during the operation are the absolute core requirements of its accuracy. A slight displacement of the positioner during the operation will easily lead to inaccurate route design, thus resulting in the failure of drilling. Therefore, it is particularly important to install an intraoperative positioning system to maintain absolute stability of the lower extremity as a prerequisite for the accuracy of the robot.

Our study found that robot navigation-assisted retrograde drilling is an advanced and effective method for the treatment of talar cartilage injuries. Compared with the arthroscopic microfracture group, patients undergone navigation-assisted retrograde drilling accepted significantly higher percentage of postoperative area reduction at 1 and 3 months. The possible explanation is that the primary structure of cartilage was preserved during the navigation-assisted retrograde drilling, which isolated the treated lesion area from the synovial fluid at an early stage. In the arthroscopic microfracture treatment group, after removal of the impaired cartilage layer, the fresh wound of microfractured talus was directly exposed to synovial fluid, the blood exudation from wound surface may recruit additional inflammatory factors. Therefore, in early stage, mesenchymal stem cells derived from the medullary cavity will received the dual effects of the physical impact of the joint fluid and the inflammatory response, which limited its early proliferation and osteogenic differentiation effects. With the progress of cartilage repair, the scar tissue and new cartilage would reconstruct on the surface of the arthroscopic microfracture wound and form a dome-like structure, which provided similar conditions for the chondrogenic transformation of mesenchymal stem cells as the navigation-assisted retrograde drilling group did. Therefore, at 6 months, the percentage of lesion repaired for both reached similar levels. The limitations of this study are as follows: small sample group, lack of power analysis (no conclusions can be made on not-statistically-significant differences), very short follow-up (it is not predictable that these results are stable over time). This study only compared and analyzed the difference in efficacy between robot-assisted retrograde drilling surgery and microfracture surgery, if the traditional retrograde drilling surgery group is added, the results of this study will be more convincing.

## 6 Conclusion

In this paper, 16 cases of early- and middle-stage OCLT in patients with or without bone cysts were included. Among them, six cases were treated by retrograde drilling for BMS and 10 cases were treated by arthroscopic microfracture surgery and all had satisfactory outcomes over a mean postoperative follow-up period of 8 months. Moreover, the follow-up revealed that with excellent efficacy and few complications, robot navigation-assisted retrograde drilling was safe and minimally invasive, and greatly reduced operative time. Consequently, robot navigation-assisted retrograde drilling for BMS was a safe and effective procedure for the treatment of OCLT.

## Data Availability

The raw data supporting the conclusions of this article will be made available by the authors, without undue reservation.
